# Cold welding of gold nanoparticles on mica substrate: Self-adjustment and enhanced diffusion

**DOI:** 10.1038/srep32951

**Published:** 2016-09-06

**Authors:** Song-Hyun Cha, Youmie Park, Jeong Woo Han, Kyeounghak Kim, Hyun-Seok Kim, Hong-Lae Jang, Seonho Cho

**Affiliations:** 1 National Creative Research Initiatives (NCRI) Center for Isogeometric Optimal Design, Seoul National University, 1 Gwanak-ro, Gwanak-gu, Seoul 088826, Republic of Korea; 2 College of Pharmacy, Inje University, 197 Inje-ro, Gimhae, Gyeongnam 621-749, Korea; 3 Department of Chemical Engineering, University of Seoul, Seoul, 130-743, Korea

## Abstract

From the images of HR-TEM, FE-SEM, and AFM, the cold welding of gold nanoparticles (AuNPs) on a mica substrate is observed. The cold-welded gold nanoparticles of 25 nm diameters are found on the mica substrate in AFM measurement whereas the size of cold welding is limited to 10 nm for nanowires and 2~3 nm for nanofilms. Contrary to the nanowires requiring pressure, the AuNPs are able to rotate freely due to the attractive forces from the mica substrate and thus the cold welding goes along by adjusting lattice structures. The gold nanoparticles on the mica substrate are numerically modeled and whose physical characteristics are obtained by the molecular dynamic simulations of LAMMPS. The potential and kinetic energies of AuNPs on the mica substrate provide sufficient energy to overcome the diffusion barrier of gold atoms. After the cold welding, the regularity of lattice structure is maintained since the rotation of AuNPs is allowed due to the presence of mica substrate. It turns out that the growth of AuNPs can be controlled arbitrarily and the welded region is nearly perfect and provides the same crystal orientation and strength as the rest of the nanostructures.

Recently, for several years, gold nanoparticles (AuNPs) have received considerable attention in various fields due to their versatile applications including drug delivery, medical diagnostics, biosensors, cancer therapeutics, and so on^1^. It is well known to have geometrical properties of metallic nanoparticles such as size and morphology that have significant impact on the structure and stability of the adsorbed biological entities as well as the nanoscale structural performances. To effectively utilize the nanoparticles for their specific purposes, it is important to grasp the characteristics and eventually control the size and morphology of nanoparticles. In this paper, we observed that the aggregation of AuNPs occurs during the preparation stage of samples for AFM (Atomic Force Microscopy) measurement, which turns out that the aggregation originates from a cold-welding phenomenon.

The cold-welding is a solid-state welding process at ambient temperature, which requires no fusion and intense energy at interfaces. Its processes are completed at room temperature and are faster than most other welding processes, without noticeable fusion occurring at the welding interfaces. As a result, during the welding process, the single-crystalline structures of the welded region were well maintained, barely leaving either defects or improper structures. In the cold-welded nanoparticles, the welding zone had the same lattice structure and connected to the original particles without any observable grain boundaries^2^. Thus, the idea of cold-welding has become an attractive process as an efficient assembly tool of nanostructures without any loss of original characteristics. It can be extensively used for the fabrication tool of elemental nanostructures such as carbon nanotubes and metal/semiconductor-filled carbon nanotubes, metal/semiconductor/ceramic nanowires and nanoparticles, and so on^2^,^3^.

The cold welding phenomena could happen in an either chemical or physical way. In the chemical way, the cold welding of AuNPs at oil/water interface was observed at room temperature by injecting ethanol and toluene into the AuNP (18 nm size) solution^4^. Ethanol changes lateral capillary attraction of interfacial nanoparticles at oil/water interface resulting in the welding of AuNPs. The cold welding was also observed by chemical methods using Fenton’s reagent to a citrate-reduced AuNPs (17 nm size)^5^. The Fenton’s reagent oxidizes citrate ions leading to the elimination of citrate from the surface which leads to the cold welding of AuNPs. The main force of cold welding is the change of lateral capillary attraction in the former and the oxidation of citrate ions by chemicals in the latter. In the physical way, the merging of silver nanoparticles (AgNPs) and AuNPs is driven by selective wettability of AgNPs, independent of their size and shape (spheres or rods)^6^. The AgNPs behaves as a soft matter, whereas AuNPs as a hard surface being wetted and retaining its original morphology. It is also reported that the cold welding of gold nanocrystals bonding in solution can occur in the pathway of defect-free bonding if their lattices aligned to within a critical angle^7^. Despite extensive work regarding the interactions between nanoparticles, the effect of interaction with substrate on the cold welding is rarely found in literature. Regarding the physics in the cold-welding of nanoscale materials, the following is experimentally known in the literature so far;

1. **Film**: If supported on compliant elastomers, thin gold films could weld together at remarkably low loads under ambient laboratory conditions^8^.
2. **Nanowire**: Single-crystalline gold nanowires with diameters between 3 and 10 nm can be cold-welded under the conditions of mechanical contact and low applied pressures^9^.
3. **Nanoparticle**: Even though numerous results are reported by chemical methods, there are no scientific reports regarding the cold welding of nanoparticles by physical actions. The misaligned nanoparticles rotate when they merge to minimize the overall surface and defect energies. The cold-welded AuNPs of 25 nm diameters are found on the mica substrate.

In macroscale bulk metals, the initiation of cold welding requires the following conditions; a high applied load, and an atomically clean, flat, and ductile surface in an ultrahigh-vacuum environment^8^. For the metallic nanoparticles, however, the cold welding can occur easily on a substrate since there are so-called ‘*self-adjustment*’ that matches crystalline orientations, requiring little external forces. Generally, the high quality of the cold welding is attributed to the followings^2^:

1. **Oriented-attachment:** The mechanical manipulation of nanowires helps to match the orientation of the samples. Matching the orientation is a key for the successful cold welding and naturally activated in the AuNPs on mica substrate.
2. **Atomic diffusion:** The use of a clean surface under conditions of high vacuum turns out to be an important factor. Also, small pressures are required for the process to occur, resulting in a fairly perfect crystal structure for the final metallic nanoparticles. The high surface to volume ratio of AuNPs enhances atomic diffusion. Just after contact of the particles started, we observed a strong tendency of welding; therefore surface atoms diffuse fast. The diffusion barrier for a single metal atom on a metal surface is known to be as low as less than 1 eV^2^,^10^. Thus, thermal activation is enough to overcome such low barriers even at room temperature but the size of the possible cold-welding is limited. The AuNPs are subjected to forces from mica substrate that causes the rigid body motion of AuNPs as well as the interaction forces.
3. **Surface relaxation and reconstruction:** The stress tensor shows a low average value during welding with oscillations indicating tension and relaxation stages. Fast Fourier transformations (FFT) from images taken from both the welded and the remaining segments of nanoparticles confirmed that the second welding zone and the remaining part of the AuNPs were both single crystalline, in the same orientation^2^.

In this paper, we discuss about the cold-welding of AuNPs on a mica substrate using HR-TEM (High Resolution – Transmission Electron Microscopy), FE-SEM (Field Emission – Scattering Electron Microscopy), and AFM images. The cold welding of AuNPs is simulated using the molecular dynamics (MD) computations of LAMMPS numerical models with and without the mica substrate. The influence of mica substrate on cold welding is explained in terms of ‘*self-adjustment*’ and ‘*enhanced diffusion*.’ Those characteristics are verified by the normal vector history of AuNPs during the self-adjustment period and by the energy considerations, continuity of lattice structures, and stacking faults to show the enhanced diffusion.

## Results and Discussions

In this paper, the AuNPs are experimentally obtained using the green synthesis^11^,^12^,^13^,^14^,^15^,^16^. When measuring the size of AuNPs in the HR-TEM and AFM images, we observe a significant difference; the AuNPs in AFM image are much bigger than those of the HR-TEM image. The mica substrate for AFM scanning is made from silicate and others. It is well-known that there is a strong attractive force between gold atoms and silicate^8^, which leads to the conjecture that something has happened in the sample on the mica substrate for the AFM scan.

### Comparison of HR-TEM and AFM (SEM) Images

**Figure 1:**
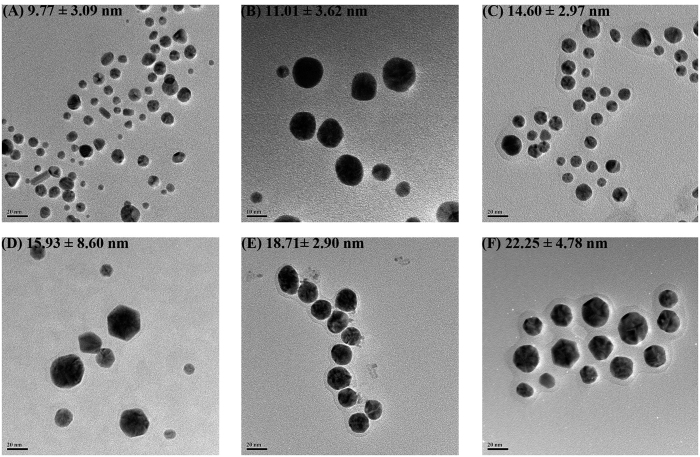
HR-TEM images of AuNPs synthesized experimentally. All AuNPs in the images were synthesized with HAuCl_4_·3H_2_O as a precursor ion. Reducing agents used were (**A**) *Polygala tenuifolia* extract, (**B**) vancomycin, (**C**) resveratrol, (**D**) gallotannin, (**E**) ampicillin and (**F**) chlorogenic acid. Note that there were no AuNPs whose diameter were bigger than 30 nm. Detailed experimental procedures for synthesizing AuNPs were described in each reference. Experimental conditions produced spherical-shaped AuNPs as shown in the images. After obtaining HR-TEM images, discrete AuNPs from the images were randomly selected to measure average diameter (nm). The number of AuNPs selected for diameter measurement is as follows. (**A**) 277, (**B**) 208, (**C**) 118, (**D**) 189, (**E**) 51 and (**F**) 111. Scale bars represent 20 nm except the image (**B**) whose scale bar is 10 nm.

Figure 1 shows the HR-TEM image of AuNPs synthesized experimentally using a natural substance as a reducing agent (Green synthesis). The details of green synthesis is found in the method section at the end of this paper. We notice that the size of AuNPs measured by HR-TEM images varies from 10 to 20 nm as shown in the histogram in the reference papers^11^,^12^,^13^,^14^,^15^,^16^. However, there were no AuNPs bigger than 30 nm in the HR-TEM images.

**Figure 2:**
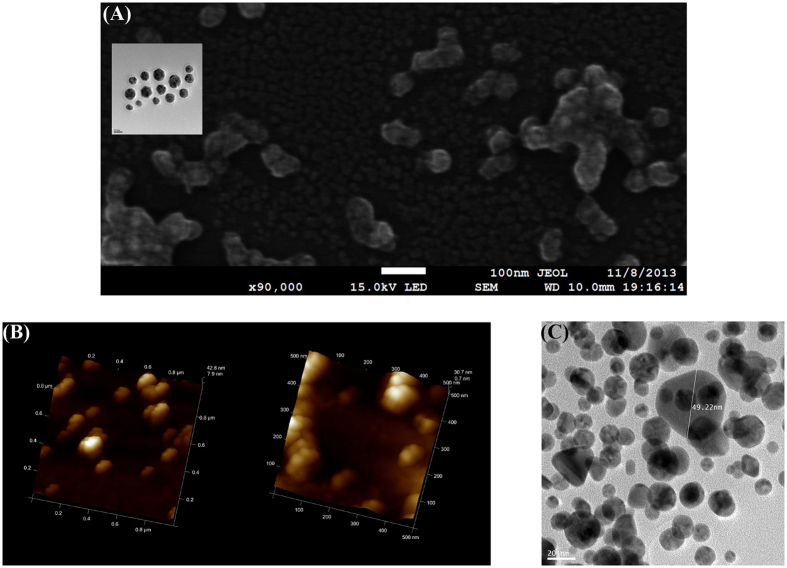
Images of AuNPs obtained from various devices. (**A**) FE-SEM image of chlorogenic acid-AuNPs after AFM scan. Average size measured in FE-SEM was found to be 59.35 ± 4.67 nm. The inset shows the corresponding HR-TEM image of chlorogenic acid-AuNPs before AFM scan with an average diameter of 22.25 ± 4.78 nm. (**B**) AFM 3D height image of chlorogenic acid-AuNPs. Scale bars represent 1 μm × 1 μm (left) and 500 nm × 500 nm (right). (**C**) HR-TEM image of resveratrol-AuNPs detached from mica substrate. Scale bar represents 20 nm. After the AFM scan of resveratrol-AuNPs on mica substrate, the AuNPs were detached from the substrate and HR-TEM image was obtained. Detailed experimental procedure of detaching resveratrol-AuNPs from mica substrate was described in experimental section. Average diameter of resveratrol-AuNPs in HR-TEM was determined to be 14.60 ± 2.97 nm as shown in Fig. 1(C). The detached AuNPs from the mica substrate were in the range of 40~50 nm and much bigger than those in HR-TEM image. The size of one nanoparticle in the image was measured as 49.22 nm.

Figure 2(B) shows the AFM height image of chlorogenic acid-AuNPs on mica substrate, which shows a diameter of 65 nm and a height of 15 nm. For the AFM sample, their sizes become bigger than 50 nm as shown in the FE-SEM image in Fig. 2(A). Compared with the AuNPs in Fig. 2(A) in the same scale, the size of AuNPs in the FE-SEM image after the AFM scanning is much bigger than that (22.25 ± 4.78 nm) of the inset HR-TEM image before the AFM scan.

As another example, the diameter of resveratrol-AuNPs before AFM scan is (14.60 ± 2.97 nm) measured by the HR-TEM image as shown in Table 1. The AFM image gives a diameter of (65.94 ± 2.26 nm) and a height of (8.69 ± 2.08 nm) which is similar to the diameter of HR-TEM measurement. After the AFM scanning, the resveratrol-AuNPs are detached from the mica substrate. For the detached particles, using the HR-TEM, their diameters are measured as (40~50 nm) in Fig. 2(c), which indicates that additional cold welding happened in the AFM sample on the mica substrate after the green synthesis. Due to the attractive forces from the mica substrate, the sphere-shaped AuNPs grow only in the plane direction parallel to the mica substrate. To investigate the phenomenon of cold welding on the mica substrate, several MD simulations are performed since the aforementioned experimental measurements provide only the images after the completion of cold welding.

The gray column indicates the measurement on HR-TEM grids right after the green synthesis. After loaded on the mica substrate, the AFM and FE-SEM measurements are sequentially performed to obtain the measurement in the white columns. Finally, detaching the AuNPs from the mica substrate, HR-TEM is performed again to obtain the re-measurement the last column.

### MD simulation of AuNPs

MD simulations are performed with LAMMPS^17^ with time steps of 0.5 fs using the velocity Verlet algorithm. 1 fs is usually used for general MD simulations. In references^18^,^19^,^20^ for MD simulation of mica, 0.5 fs is suggested. We have tested both ones to find that 0.5 fs is more stable. A Nose-Hoover thermostat is introduced to keep the temperature at 300 K in the canonical ensemble. The temperature damping constant for the Nose-Hoover thermostat is determined as 0.01 based on the reference^21^. If the constant is too big, the canonical distribution will be achieved after very long simulation times. On the other hand, too small values could result in high-frequency temperature oscillations. The temperature keeps at 300 K during the MD simulations since the solution sample of AuNPs is dried on mica at room temperature in real experiments. The interatomic potential employed in the simulations is described by the embedded atom method (EAM)^22^ where the potential is described by a pair potential and a function of the electronic density. The MD methods and the numerical model for the mica substrate are constructed, based on the references^23^,^24^,^25^.

**Figure 3:**
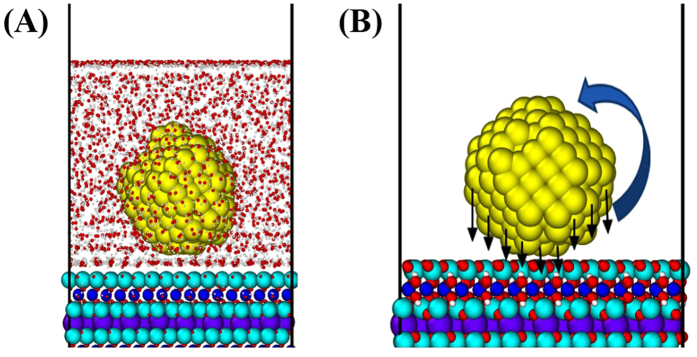
MD Simulation of AuNP on mica substrate (Supplement #1). (**A**) Rigid body motion of AuNP influenced by water molecules, (**B**) Rigid body rotation of AuNP by non-uniform attractive forces from the surface of AuNP. Water molecules obstruct the interactions between the AuNPs and the mica substrate and help to float the AuNPs. The non-uniform attractive forces from the surface of the AuNPs could result in rigid body rotation of the AuNPs. These make the AuNPs on the mica substrate rotate more vigorously, compared to the case of AuNPs without the mica substrate.

Water molecules obstruct the interactions between the AuNPs and the mica substrate and help to float the AuNPs, which leads to the easy rigid body motion of AuNP in Fig. 3(a). Also, the non-uniform attractive forces from the surface of the AuNP could result in rigid body rotation of the AuNP as shown in Fig. 3(b).

Nanoparticles are able to freely rotate compared with nanowires and nanofilms, and thus the cold welding goes along to adjust the lattice structures of the nanoparticles. It is known that the maximum size of cold welding is limited to 10 nm for nanowires^2^ and 2~3 nm for nanofilms^8^ as reported in the literature. However, in this paper, 25 nm cold-welded AuNPs are found on the mica substrate, due to the self-adjustment characteristics. To verify the ‘*self-adjustment*’ and ‘*enhanced diffusion*’ characteristics of nanoparticles on the mica substrate, two types of numerical models are constructed for the MD simulations.

- **M models** – two cases of AuNPs on mica substrate (diameters of 2.040 nm and 3.672 nm).
- **N models** – two cases of AuNPs without substrate (diameters of 2.040 nm and 3.672 nm).

The numbers of atoms in the 2.040 nm and 3.672 nm AuNPs are 762 and 1,400, respectively. Also, the number of atoms for the mica substrate is 10,752 and the millions of time steps are required. If the diameter of the AuNPs is increased to 10~20 nm, the size of mica substrate should be increased accordingly and, thus, the computing time increases prohibitively. However, several quantitative simulations are performed with the smaller M and N models, which can be handled by the current computing power.

### Self-adjustment

**Figure 4:**
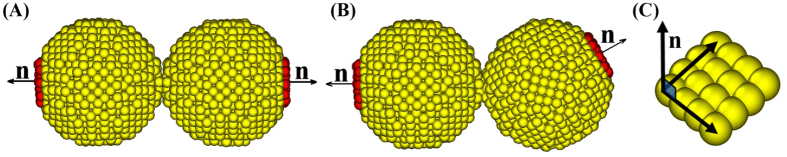
Surface normal vectors. (**A**) Initially parallel model, (**B**) Initially 30 degree rotated model, (**C**) Outward normal vector of lattice surface.

Potential energy for gold atoms can be divided into an EAM (Embedded Atom Method) potential contribution for gold and L-J (Lennard-Jones) potential for the mica substrate, *U* = *U*^*EAM*^ + *U*^*LJ*^. It is observed that the potential energy in gold atoms near the mica is bigger than that far away from the mica since the L-J potential is dependent on the interatomic distance. The EAM potential for gold nanoparticles and the L-J potential for mica substrate system are available in literature and LAMMPS code. However, the L-J parameters between the atoms of different kind for gold-mica interactions are not generally available. Based on the Lorentz-Berthelot mixing rule, we had to use a geometric average for the energy depth and an arithmetic average for the collision diameter. Thus, the qualitative comparison of numerical results with experimental ones gets better meaning than quantitative comparison. Two configurations of AuNPs are considered as shown in Fig. 4, initially parallel model (A) and initially rotated model (B).

### Initially parallel model

M and N models have a diameter of 2.040 nm and are aligned to match the lattice structures. Two outward normal vectors are defined in Fig. 4(C). The normal vectors are constructed using the averaged position data of atoms in the same plane. To obtain a unit normal vector **n** for the surface of the lattice, an equation for the plane red-colored in Fig. 4(A,B) should be determined. Using the least square regression method, we obtain the equation, from which the corresponding normal vector is determined. The inner product of normal vectors in the AuNPs can be used to measure the degree of self-adjustment. The value of 1 represents the perfectly parallel alignment of lattice surfaces. Details about the determination of the normal vector are discussed in the methods section.

**Figure 5:**
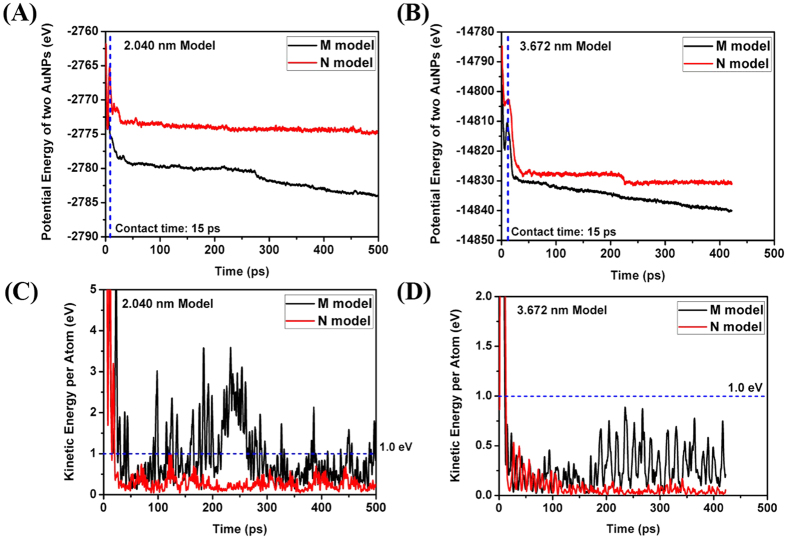
Comparison of mechanical energy per atom (Initially parallel). (**A**) Potential energy history of 2.04 nm diameter model; (**B**) Potential energy history of 3.67 nm diameter model; (**C**) Kinetic energy history of 2.04 nm diameter model; (**D**) Kinetic energy history of 3.67 nm diameter model.

Comparing the potential energy of the models, the M model has a history of smaller values than the N model as shown in Fig. 5(A). It can be inferred that the difference of potential energy is devoted to the cold welding. Notice that the M model has smaller potential energy regardless of the diameter of AuNPs and is in more stable state on the mica substrate.

Comparing the kinetic energy of one gold atom, the M model has a history of bigger values than the N model as shown in Fig. 5(C). As increasing the diameter of AuNPs, we can observe a decreasing tendency of kinetic energies as well as its difference in Fig. 5(D). The required energy per atom for cold welding is known as 1 eV. In Fig. 5(C), after the contact of two nanoparticles, many points bigger than 1 eV are observed in the M model but few points in the N model. However, as increasing the diameter of nanoparticles, the points bigger than 1 eV are rarely found in the M model and none in the N model after the contact of two nanoparticles as shown in Fig. 5(D). Thus, the cold welding could occur only on the contact surfaces of the nanoparticles. During the AFM scan in real experiment, the cold welding happens in the nanoparticles of 25 nm diameter. In this simulation, we performed the qualitative comparison of numerical results with experimental ones due to the use of averaged L-J parameters between the atoms of different kind for gold-mica interactions. Notice that the difference of kinetic energy in the M and N models decreases as we increase the diameter of AuNPs, which implies the decrease of available kinetic energy to overcome the energy barrier for cold welding. Therefore, it can also be inferred that the cold welding hardly happens if the diameter of AuNPs is bigger than a specific one.

### Initially rotated model by 30 degrees

**Figure 6:**
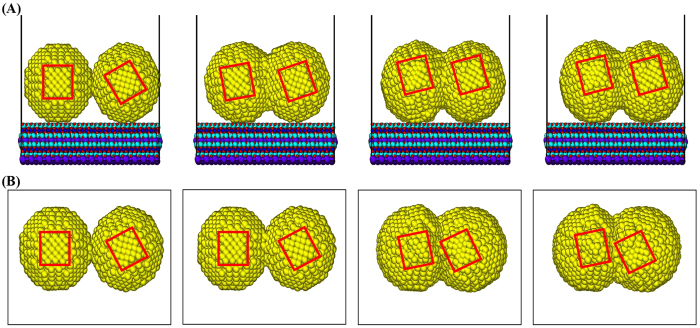
Comparison of self-adjustment (Supplement #2). (**A**) AuNPs on the mica substrate, (**B**) AuNPs without mica substrate. The AuNPs on the mica substrate rotate to align the lattice structures after the completion of cold welding. On the other hand, if the mica substrate is absent, the AuNPs do not rotate and the regular lattice structure disappears as the cold welding progresses.

To further investigate the characteristics of self-adjustment in the cold welding of AuNPs on mica substrate, we consider a case of initially rotated AuNPs by 30 degrees as shown in Fig. 4(B). The boxed regions represent the planes in the same normal direction and their in-plane rotation indicates the rotation of normal surfaces of AuNPs. As the cold welding goes along as shown in Fig. 6(A), the AuNPs on the mica substrate rotate to align the lattice structure of contact surfaces after 500 ps (check the alignment of two red boxes). This is due to the tendency to maintain the regular lattice structure around the welded region if the rotation of AuNPs is allowed. However, if the mica substrate is absent as shown in Fig. 6(B), the AuNPs do not rotate and the regular lattice structure disappears as the cold welding progresses.

**Figure 7:**
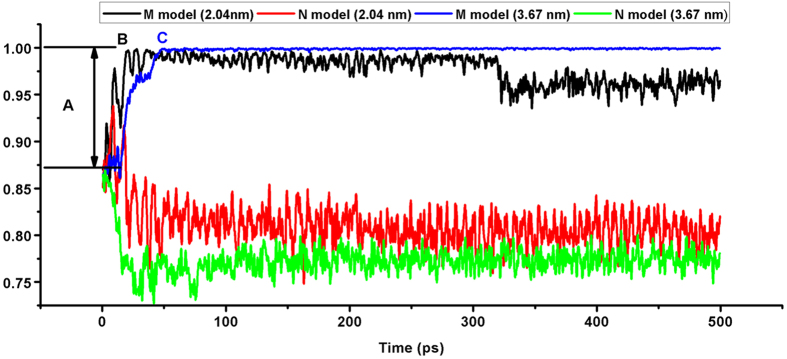
Inner product history of normal vectors. The inner product history of two M models is shown in black for the 2.040 nm model and in blue for the 3.672 nm model. Also, the inner product history of two N models is shown in red for the 2.040 nm model and in green for the 3.672 nm model. For the case of 2.040 nm M model (Black) after the contact of AuNPs, several large rotations like A occur to align the orientation of lattice structures until the AuNPs reach B region. For the case of 3.672 nm M model (Blue), the AuNPs are gradually rotated to align the orientation of lattice structures until the AuNPs reach C region.

In each of the M and N models, we construct two models whose diameters are 2.040 and 3.672 nm. The inner product of the two normal vectors in each of the four models is 0.866 since the normal vectors are initially rotated by 30 degrees. Figure 7 shows the inner product history of two M models which is shown in black for the 2.040 nm model and in blue for the 3.672 nm model. Figure 7 includes the inner product history of two N models which is shown in red for the 2.040 nm model and in green for the 3.672 nm model.

The inner products of the two M models (Black and Blue) are quickly recovered to 1.0 whereas those of the two N models (Red and Green) maintain the initial inner product values. For the case of 2.040 nm M model (Black) after the contact of AuNPs, several large rotations like A occur to align the orientation of lattice structures until the AuNPs reach B region. After that, the cold welding could continue since the amplitude of inner product implies the relative rotation of two nanoparticles. For the case of 3.672 nm M model (Blue), the AuNPs are gradually rotated to align the orientation of lattice structures until the AuNPs reach C region. After that, the relative rotation of two nanoparticles seems negligible, which implies that no further cold welding could occur. On the other hand, cold welding could continue without significantly changing the initial angle.

**Figure 8:**
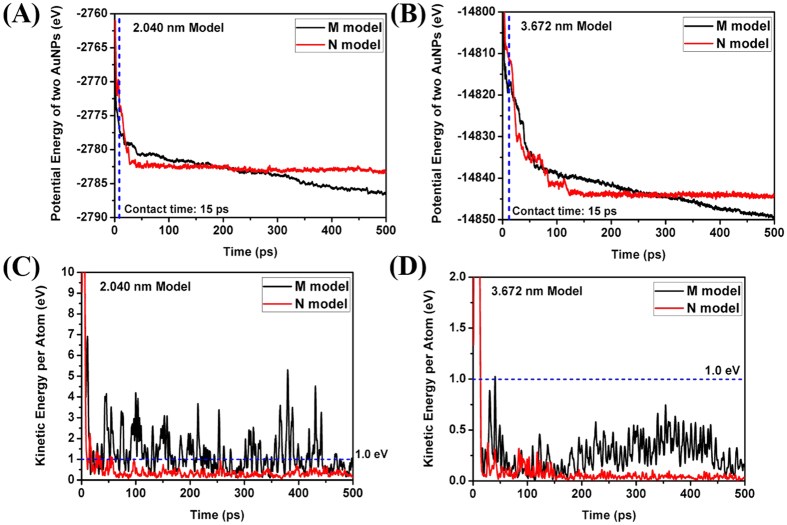
Comparison of mechanical energy per atom (Initially rotated by 30 degrees). (**A**) Potential energy history of 2.040 nm model, (**B**) Potential energy history of 3.672 nm model, (**C**) Kinetic energy history of 2.040 nm model, (**D**) Kinetic energy history of 3.672 nm model.

Comparing the potential energy of the models, the M model has a history of smaller values than that of the N model as shown in Fig. 8(A).

Comparing the kinetic energy of one gold atom, the M model has a history of bigger values than that of the N model as shown in Fig. 8(C). After the contact of two AuNPs, many points bigger than 1 eV are observed in the M model but few points in the N model. As increasing the diameter of the AuNPs, we can observe a decreasing tendency of kinetic energies as well as its difference in Fig. 8(D). The points bigger than 1 eV are rarely found in the M model and none in the N model after the contact of two AuNPs.

### Enhanced diffusion

In order to analyze the results of cold welding mechanism, the following methods^10^ were used,

- Ackland-Jones method.
- Centro-symmetry parameter method.

The Ackland-Jones method^26^ is essentially an heuristic algorithm that compares the angular distribution of a perfect crystalline lattice as well as the lattices with small distortions generated within the simulations, attributing either fcc (face-centered cubic), bcc (body-centered cubic), hcp (hexagonal close packed), or ico (icosahedral) structures. The centro-symmetry parameter^27^ is a largely used method to identify defects in crystals such as stacking faults in fcc structures. The centro-symmetry analysis shows once again that the cold welding occurred with low stress, and at the end of the process, a crystalline structure with very few defects was achieved, recovering most of the original characteristics of the pristine AuNPs.

**Figure 9:**
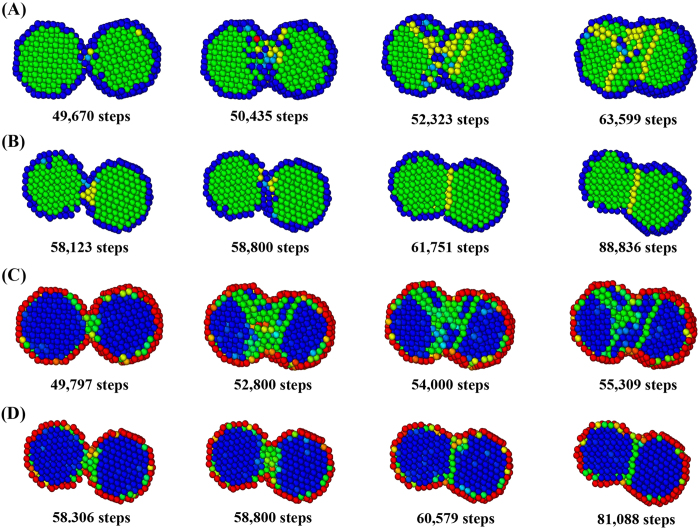
Comparison of various measures for the quality of cold welding. (**A**) Ackland-Jones parameter contour in M model, (**B**) Ackland-Jones parameter contour in N model, (**C**) Centro-symmetry parameter contour in M model, (**D**) Centro-symmetry parameter contour in N model. Bcc and hcp structures (**A**) and stacking faults (**C**) are expanding from the welding surface as the cold welding progresses in M model. After some relaxation period, fcc structure is finally recovered. On the other hand, a small portion of hcp structures (**B**) and stacking faults (**D**) near the welding surface are generated around the welding surface as the cold welding progresses in N model. After some relaxation period, the fcc structure is quickly recovered. The region of atomic diffusion is very limited, which could lead to incomplete welding.

We showed that the welded AuNPs retain their crystalline fcc structure even in the welded region, in agreement with the known experimental findings^2^ that the few defects introduced in the process reconstruct to restore the fcc structure. To measure the regularity of lattice structures, consider the history of Ackland-Jones parameter during the cold welding. In the M model of Fig. 9(A), the following colors are assigned to the various lattice structures; blue for unknown, sky blue for bcc, green for fcc, yellow for hcp, and red for ico. Figure 9(A) shows that bcc and hcp structures are expanding from the welding surface as the cold welding progresses. After some relaxation period, fcc structure is finally recovered. On the other hand, in the N model of Fig. 9(B), a small portion of hcp structures is generated around the welding surface as the cold welding progresses. After some relaxation period, the fcc structure is quickly recovered. The region of atomic diffusion is very limited, which could lead to incomplete welding.

To measure the quality of lattice structures, consider the history of centro-symmetry parameter during the cold welding. Figure 9(C) shows that stacking faults expand from the welding surface as the cold welding progresses. After some relaxation period, the regular fcc structure is finally recovered. On the other hand, in the N model of Fig. 9(D), stacking faults occur near the welding surface as welding progresses. After relaxation period, the regular fcc structure is quickly recovered. The region of atomic diffusion is very limited, which could lead to incomplete welding.

### Simulation of cold welding process

**Figure 10:**
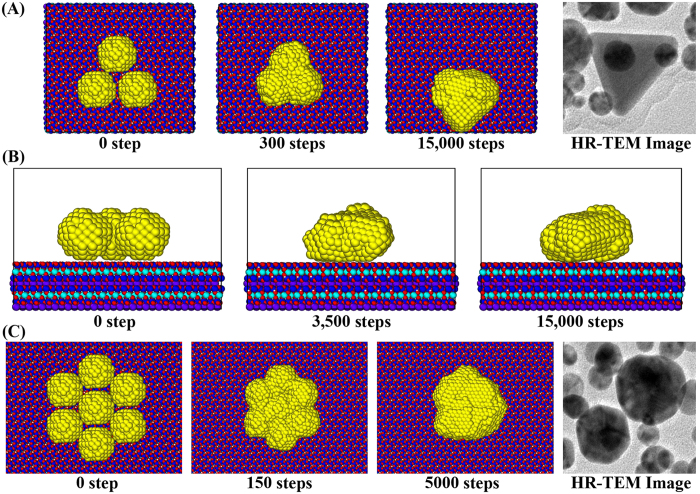
MD snapshots of AuNPs on mica substrate (Supplement #3, #4, #5). (**A**) Top view of 3 AuNPs on the mica substrate, (**B**) Side view of 3 AuNPs on the mica substrate, (**C**) Top view of 7 AuNPs on the mica substrate. The last pictures in Fig. 10(A,C) shows the HR-TEM images of the resveratrol-AuNPs detached from the sample on the mica substrate after AFM scan. The AuNPs grow in the plane parallel to the mica substrate, maintaining regular lattice structures as shown in Fig. 10(B). As a consequence of the cold welding, it is observed that the regularity of lattice structure is well maintained.

The HR-TEM images of the AuNPs in Fig. 10(A,C) are obtained from single HR-TEM sample of AuNPs detached from the mica substrate after the AFM scan, in other words, after the completion of cold welding. For the simulation of the cold welding process, the numerical model in Fig. 10(A) consists of 3 AuNPs on the mica substrate made of 16 × 8 × 1 unit cells. We can notice that the linear side of the triangle is not generated from the clustering of gold atoms during the green synthesis but could be constructed through the cold welding process.

After the MD simulations, the shape of AuNPs indicates that the height of cold-welded AuNPs is 1.9 nm which is similar to the diameter (1.8 nm) of original AuNPs as shown in Fig. 10(B). The AuNPs grow in the plane parallel to the mica substrate, maintaining regular lattice structures. As a consequence of the cold welding, it is observed that the regularity of lattice structure is well maintained.

For the case of 7 nanoparticles in Fig. 10(C), the hexagonal nanoparticles are not generated from the clustering of gold atoms during the green synthesis but could be constructed through the cold welding process. In the cold welding process, the AuNPs are going through the iterative process of clustering and migration until they are sufficiently stabilized as shown the history of potential energy in the AuNPs in Figs 5 and 7.

## Conclusions

Due to the interactions with mica substrate, it can be inferred that the cold welding of AuNPs on the mica happens in in-plane direction, based on the observation that the diameter of AuNPs in the AFM image is much bigger than those in the HR-TEM image and the height of AuNPs in the AFM image is similar to the diameter of HR-TEM measurement. It is known that the maximum size of cold welding is limited to 10 nm for nanowires and 2~3 nm for nanofilms. However, 25 nm cold-welded AuNPs are found on mica substrates. The AuNPs is able to freely rotate, compared with nanowires and nanofilms, the cold welding goes along adjusting the lattice structure of AuNPs. Matching the orientation is a key to realizing successful welding naturally activated in AuNPs on mica substrates. Also, as the water molecules in the AFM sample evaporate, the potential energy from the mica substrate and the kinetic energy of AuNPs provide sufficient energy to overcome the diffusion barrier of gold atoms. The cold-welded region is nearly perfect and consequently provides the same crystal orientation and strength as the rest of nanostructures.

The potential energy difference due to the presence of mica substrate results in the increase of kinetic energy, which can be observed in Figs 5 and 8. Through MD simulations, the increased mobility can be observed in Fig. 6 and animation (supplement 2), which finally leads to the better quality of cold welding in terms of Ackland-Jones and centro-symmtry parameters in Fig. 9. As a consequence of cold welding, the regularity of lattice structure is maintained in the model with mica substrate. This is due to the tendency to maintain the regular lattice structure around the welded region if the rotation of AuNPs is allowed due to the presence of mica substrate. As the cold welding progresses, bcc and hcp structures as well as stacking faults expand from the welding surface. After some relaxation period, a regular fcc structure is finally recovered.

The attractive features of cold-welding in this paper are two-fold. *First*, it joins nanostructures without intense heating or other external forces. The cold-welded region is nearly perfect and consequently provides the same crystal orientation and strength as the rest of the nanostructures. *Second*, the growth of AuNPs can be controlled in the direction of interest. The linear side of triangle, hexagonal, and oval shapes in Fig. 10 can be constructed through the cold welding process. These features can be extensively utilized in the fabrication of various nanostructures.

## Methods

### Experiment

For the synthesis of AuNPs, gold (III) chloride trihydrate (HAuCl_4_·3H_2_O) was purchased from Sigma-Aldrich (St. Louis, MO, USA). Resveratrol-AuNPs were synthesized according to our previous report^13^. Resveratrol-AuNPs were loaded onto a mica substrate (grade V-1, 25 mm × 25 mm length, 0.15 mm thick, SPI Supplies Division of Structure Probe, West Chester, PA, USA) and dried overnight in a 37 °C oven. After drying overnight, deionized water was put on the sample-loaded mica and the resveratrol-AuNPs was detached from the mica substrate. Then, the removed resveratrol-AuNPs was pipetted onto a carbon-coated copper grid (carbon type-B, 300 mesh copper grid, Ted Pella, Inc., Redding, CA, USA) and the sample-loaded grid was air-dried. A JEM-3010 microscope operated at 300 kV (JEOL Ltd., Tokyo, Japan) was utilized to get HR-TEM images.

### MD simulation

All MD simulations are performed by utilizing a public domain code, LAMMPS^17^ (Large-scale Atomic/Molecular Massively Parallel Simulator). To construct a water pressure model, a simulation setup tool Packmol^28^ (open source) is used. Using the Packmol, water molecules are randomly placed into a cube with the size of 60 by 60 by 60 Angstroms. The determined number of water molecules in the cube is 1863, based on the density (1 g/cm^3^) of water liquid since the experimentally synthesized AuNPs are in solution. After randomly packing molecules, an energy minimization of corresponding system is conducted with a steepest descent algorithm. Furthermore, an isothermal (NVT) simulation is conducted for 1 ps at 358 K (85 °C in oven) with the time step of 0.5 fs in accordance to the real experiment to speed up the drying process. It is known that the drying temperatures of 300 K and 358 K did not affect the resulting size of AuNPs in ref. 13.

### Interatomic potentials

The bonded and non-bonded interactions of oxidized Caffeic acids are described by the general AMBER force field (GAFF)^29^ as shown in Eq. (1).


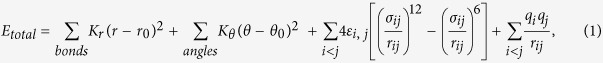


The bonded interaction parameters *K*_*r*_ and *K*_*θ*_ are force constants. *r*_0_ and *θ*_0_ are the equilibrium bond length and angle, respectively. For the non-bonded interactions, the Lennard-Jones (L-J) parameters of *ε* and *σ* are introduced to describe repulsive and attractive contributions. The last term in Eq. (1) stands for the Columbic pairwise interaction where *q* is the charge of atom. The water molecules were described using the TIP3P model^30^ with a long-range Columbic solver. Force field parameters to model the mica substrate are obtained from relevant literature^18^,^19^,^20^.

To describe the interactions between gold-gold, we employ an embedded atom method (EAM) potential. For non-bonded interactions between different types of atoms, Lorentz-Berthelot mixing rules are used to calculate *ε*_*ij*_ and *σ*_*ij*_ as Eqs (2) and (3).


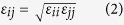


and


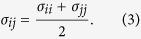


The interatomic potentials between gold and oxygen/hydrogen atoms are considered using the L-J potential parameters^30^. The atom of single kind has unique L-J parameters such as collision diameter σ and energy depth ε. The L-J parameters between the atoms of different kind is not generally available. Thus, based on the Lorentz-Berthelot mixing rule, we use a geometric average for the energy depth (eq. 2) and an arithmetic average for the collision diameter (eq. 3).

### Modelling of mica

Muscovite (KAl_2_(Si_3_Al)O_10_(OH)_2_) belongs to the group of 2:1 layer silicates and consists of an [AlO_6_] octahedral sheet sandwiched between two [(Si, Al)O_4_] tetrahedral sheets. The monoclinic C2/c 2M1 muscovite crystal structure was used as the initial input structure for our MD simulations^31^. The experimental lattice optimizations are a = 5.20˚A, b = 8.99˚A, c = 20.03˚A, alpha = 90˚, beta = 94.50˚ and gamma = 90.00˚. The muscovite surface was built by cleaving the structure along the (0 0 1) plane at the middle of the inter-layer space^23^,^24^,^25^. The AuNPs are attached to the surface of mica substrate by the exchange mechanism of potassium metal ion and AuNPs. In this paper, the potassium metal ion on the surface is not considered and MD simulations are performed with the AuNPs located on the mica substrate. The necessary potential parameters for the mica substrate are obtained from references^18^,^19^,^20^. Periodic boundary conditions are imposed in x and y directions. We construct the unit cell of mica substrate that can be extensively used according to the size and number of AuNPs. The mica slab is relaxed at 358 K during 5,000 steps before putting the mica slab and the AuNPs together. After 5,000 steps, the potential energy and kinetic energy are in stable state. The MD simulations are performed under the condition that atoms within 1 nm from the bottom are fixed and the others are free to move.

### Determination of normal vector

For the equation of arbitrary plane *z* = *ax* + *by* + *c* and the position **x**_*i*_ = (*x*_*i*_, *y*_*i*_, *z*_*i*_) of atom *i* in the surface of the lattice, we define the following measure G that represents the vertical difference of all atoms in the surface,


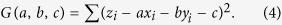


Using 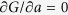, 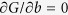, and 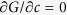 to minimize Eq. (4), we obtain the following,


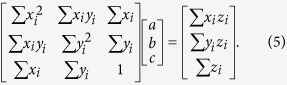


The unit normal vector is obtained from the solution of Eq. (5).


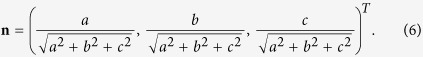


The inner product of normal vectors in the AuNPs can be used to measure the degree of self-adjustment. The value of 1 represents the perfectly parallel alignment of lattice surfaces.

## Additional Information

**How to cite this article**: Cha, S.-H. *et al.* Cold welding of gold nanoparticles on mica substrate: Self-adjustment and enhanced diffusion. *Sci. Rep.* **6**, 32951; doi: 10.1038/srep32951 (2016).

## Supplementary Material

Supplementary Information

Supplementary Movie 1

Supplementary Movie 2

Supplementary Movie 3

Supplementary Movie 4

Supplementary Movie 5

Supplementary Movie 6

Supplementary Movie 7

## Figures and Tables

**Table 1:** Size comparison of AuNPs from various devices (unit:nm).

Reducing Agent	*HR-TEM	AFM		FE-SEM	HR-TEM (Remeasurement)
		Diameter	Height		
*polygala tenuifolia* extract	9.77 ± 3.09	198 ± 37.5	20.1 ± 7.06	—	—
vancomycin	11.0 ± 3.62	30.3 ± 3.83	10.8 ± 2.21	—	—
resveratrol	14.6 ± 2.97	65.9 ± 2.26	8.69 ± 2.08	—	40~50
gallotannin	15.9 ± 8.60	62.9 ± 6.57	9.29 ± 2.16	69.7 ± 6.57	—
ampicillin	18.7 ± 2.90	37.4 ± 6.74	9.97 ± 3.70	40.2 ± 4.59	—
chlorogenic acid	22.3 ± 4.78	52.0 ± 15.2	9.70 ± 5.31	59.4 ± 4.67	—
