# Supplementary material for: Cold welding of gold nanoparticles on mica substrate: Self-adjustment and enhanced diffusion
Source: Sci Rep. 2016 Sep 6;6:32951. doi: 10.1038/srep32951 (PMC5011739; doi:10.1038/srep32951)
Supplement: Supplementary Information [file srep32951-s1.doc]

***Description of video files***

**TITLE:** Cold welding of gold nanoparticles on mica substrate: Self-adjustment and enhanced diffusion

**AUTHOR NAMES:** Song-Hyun Cha, Youmie Park, Jeong Woo Han, Kyeounghak Kim, Hyun-Seok Kim, Hong-Lae Jang, Seonho Cho

**List of Supplementary Videos:**

1. Floating and rotation of one nanoparticle

2. Comparison of self-adjustment with and without mica substrate

3. MD simulation of three nanoparticles (Top view)

4. MD simulation of three nanoparticles (Side view)

5. MD simulation of seven nanoparticles

**[Supplement #1]** **Floating and rotation of one nanoparticle**


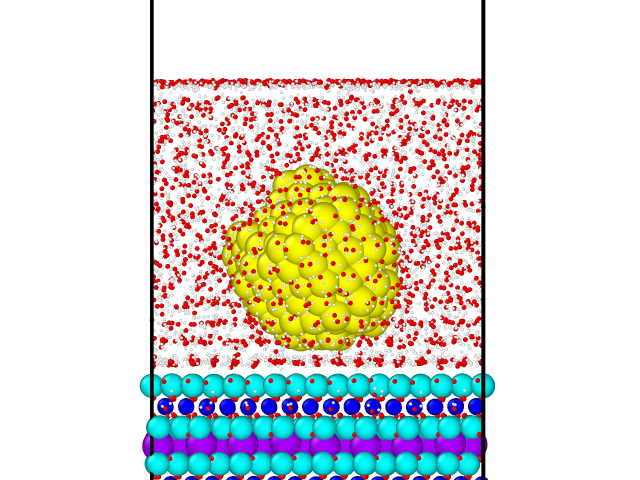


**Nanoparticle in water**: Water molecules (red dots) between the AuNP and the mica substrate obstruct the interactions and help to float the AuNP, which leads to the easy rigid body motion of AuNP.


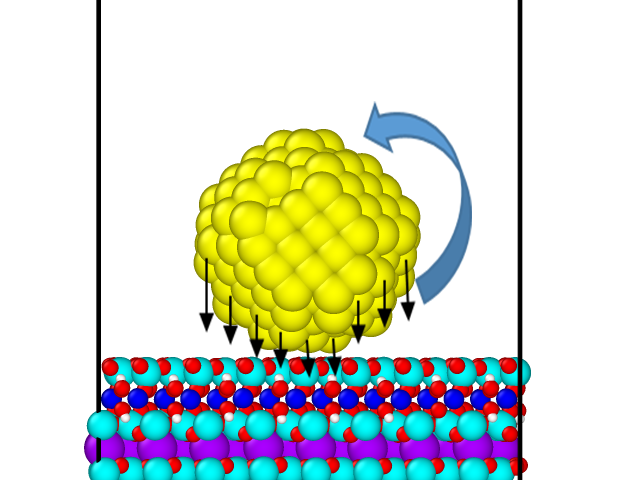


**Rotation of nanoparticle**: The non-uniform attractive forces on the surface of AuNP could result in the rigid body rotation of AuNP. These make the AuNPs on the mica substrate rotate more vigorously, compared to the case of AuNPs without the mica substrate. (The image of water molecules are removed for better visibility.)

**[Supplement #2]** **Comparison of self-adjustment with and without mica substrate**


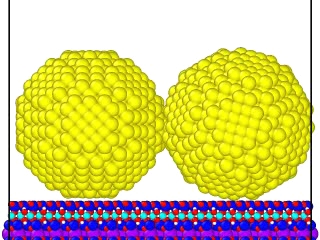

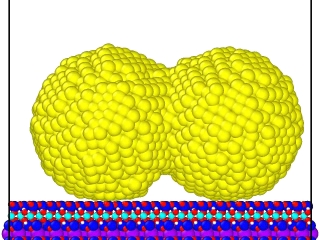


Initial After cold weding

**Mica**: To further investigate the characteristics of self-adjustment in the cold welding of AuNPs on mica substrate, we consider a case of initially rotated AuNPs by 30 degrees. As the cold welding goes along, the AuNPs on the mica substrate rotate to align the lattice structures after the completion of cold welding. This is due to the tendency to maintain the regular lattice structure around the welded region if the rotation of AuNPs is allowed.


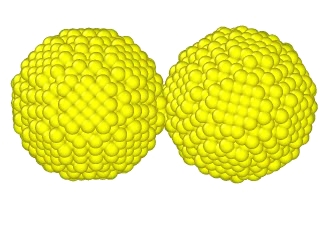

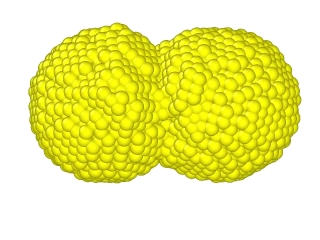


Initial After cold weding

**No mica**: The AuNPs do not rotate and the regular lattice structure disappears as the cold welding progresses.

**[Supplement #3]** **MD simulation of three nanoparticles (Top view)**


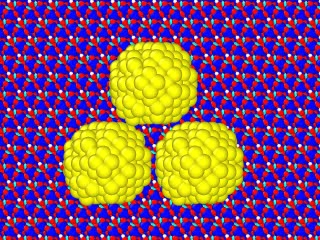

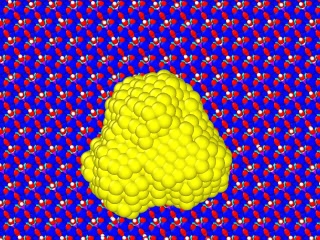


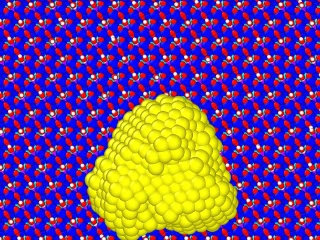

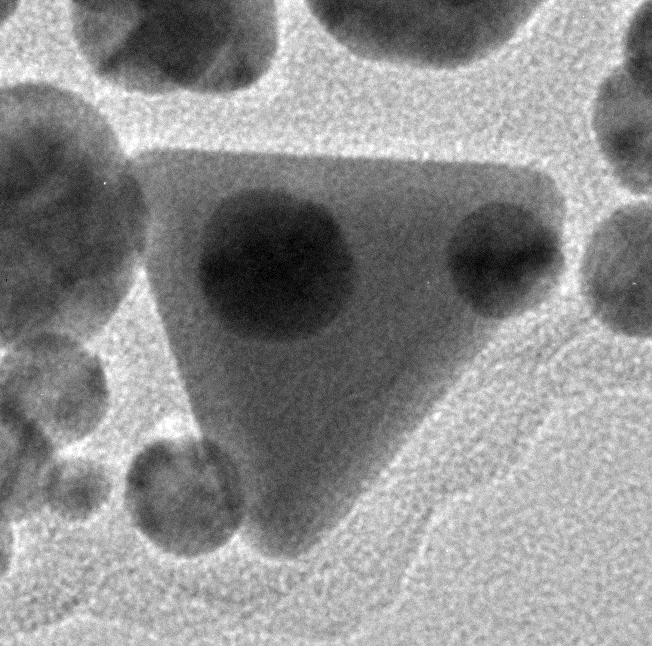


**Top view**: For the simulation of the cold welding process, the numerical model consists of 3 AuNPs on the mica substrate made of 16×8×1 unit cells. We can notice that the linear side of the triangle could be constructed through the cold welding process. The linear side of the triangle is not generated from the clustering of gold atoms during the green synthesis but could be constructed through the cold welding process.

**[Supplement #4]** **MD simulation of three nanoparticles (Side view)**


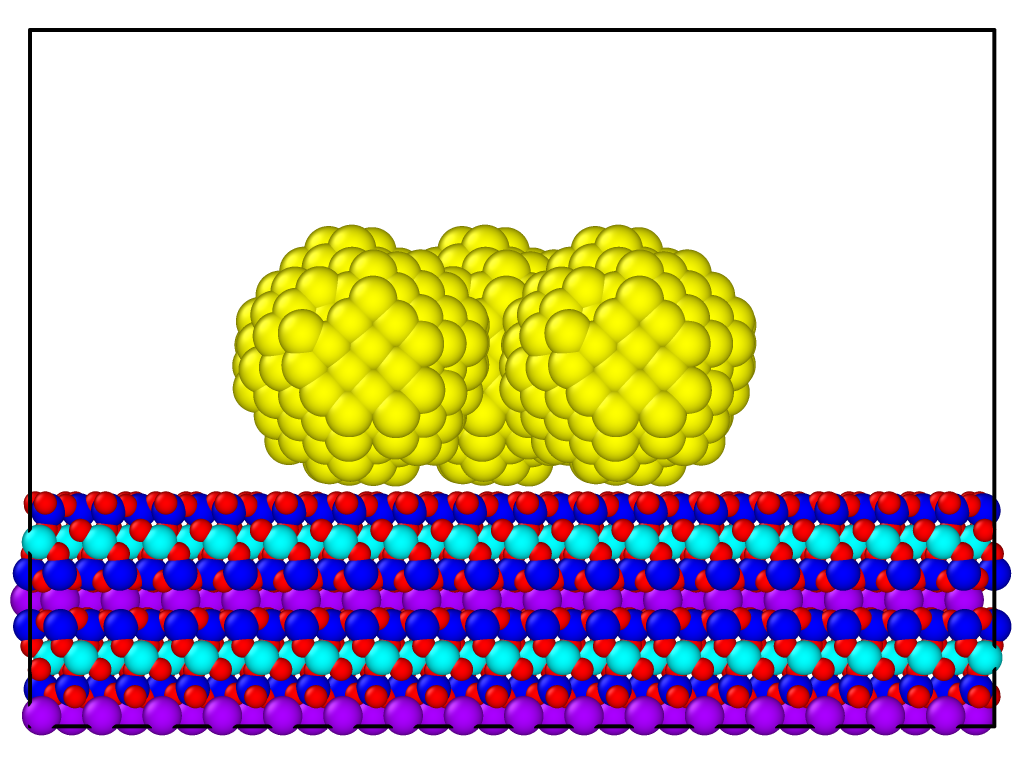

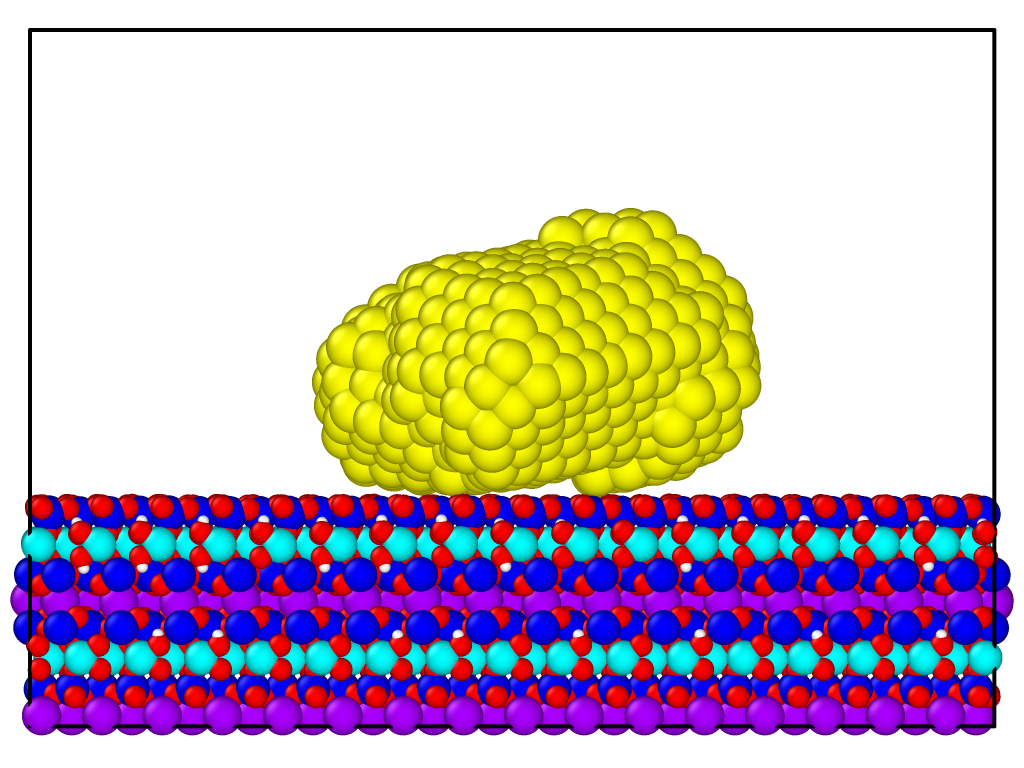


**Side view**: After equilibration, the shape of AuNPs indicates that the height of cold welded AuNPs is similar to the diameter of original AuNPs. We notice that The AuNPs grow in the plane parallel to the mica substrate, maintaining regular lattice structures. As a consequence of the cold welding, it is observed that the regularity of lattice structure is well maintained.

**[Supplement #5]** **MD simulation of seven nanoparticles**


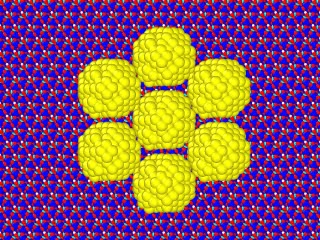

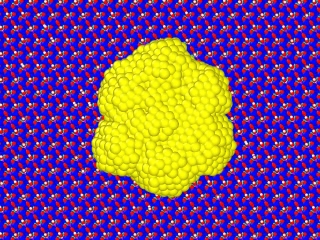


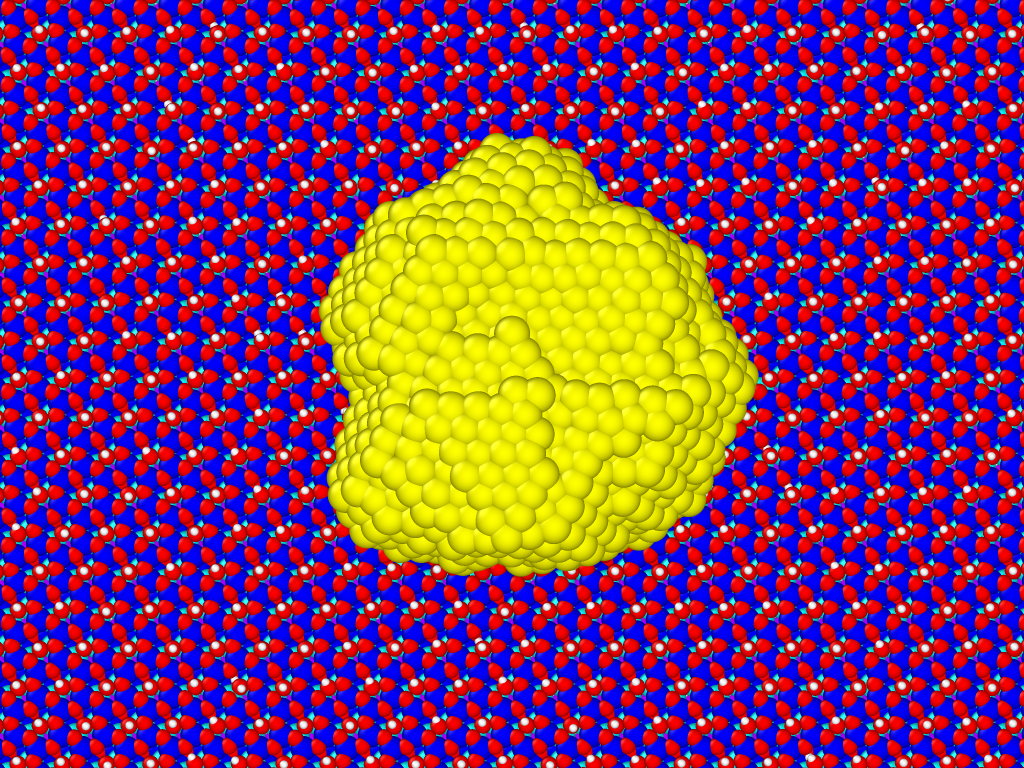

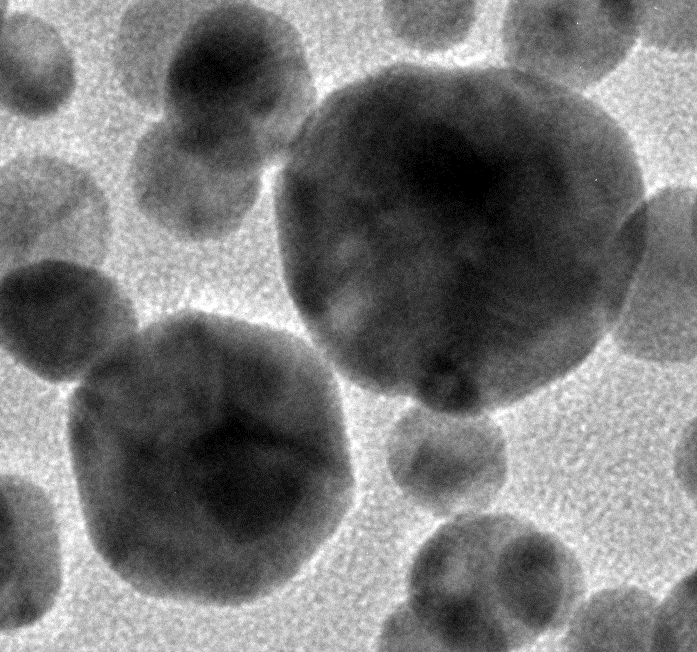


**Top view**: The hexagonal nanoparticles are not generated from the clustering of gold atoms during the green synthesis but could be constructed through the cold welding process. In the cold welding process, the nanoparticles are going through the iterative process of clustering and migration until they are sufficiently stabilized.
